# Leveraging Existing Cohorts to Study Health Effects of Air Pollution on Cardiometabolic Disorders: India Global Environmental and Occupational Health Hub

**DOI:** 10.1177/1178630220915688

**Published:** 2020-04-20

**Authors:** Gagandeep K Walia, Siddhartha Mandal, Suganthi Jaganathan, Lindsay M Jaacks, Nancy L Sieber, Preet K Dhillon, Bhargav Krishna, Melina S Magsumbol, Kishore K Madhipatla, Dimple Kondal, Richard A Cash, K Srinath Reddy, Joel Schwartz, D Prabhakaran

**Affiliations:** 1 Public Health Foundation of India, New Delhi, India; 2 Centre for Chronic Disease Control (CCDC), New Delhi, India; 3 Department of Global Health and Population, Harvard T.H. Chan School of Public Health, Boston, MA, USA; 4 Department of Environmental Health, Harvard T.H. Chan School of Public Health, Boston, MA, USA; 5 Department of Epidemiology, London School of Hygiene and Tropical Medicine, London, UK

**Keywords:** Air pollution, cardiovascular diseases, cohort studies, India, particulate matter

## Abstract

Air pollution is a growing public health concern in developing countries and poses a huge epidemiological burden. Despite the growing awareness of ill effects of air pollution, the evidence linking air pollution and health effects is sparse. This requires environmental exposure scientist and public health researchers to work more cohesively to generate evidence on health impacts of air pollution in developing countries for policy advocacy. In the Global Environmental and Occupational Health (GEOHealth) Program, we aim to build exposure assessment model to estimate ambient air pollution exposure at a very fine resolution which can be linked with health outcomes leveraging well-phenotyped cohorts which have information on geolocation of households of study participants. We aim to address how air pollution interacts with meteorological and weather parameters and other aspects of the urban environment, occupational classification, and socioeconomic status, to affect cardiometabolic risk factors and disease outcomes. This will help us generate evidence for cardiovascular health impacts of ambient air pollution in India needed for necessary policy advocacy. The other exploratory aims are to explore mediatory role of the epigenetic mechanisms (DNA methylation) and vitamin D exposure in determining the association between air pollution exposure and cardiovascular health outcomes. Other components of the GEOHealth program include building capacity and strengthening the skills of public health researchers in India through variety of training programs and international collaborations. This will help generate research capacity to address environmental and occupational health research questions in India. The expertise that we bring together in GEOHealth hub are public health, clinical epidemiology, environmental exposure science, statistical modeling, and policy advocacy.

## Background

Air pollution is a growing concern in developing countries contributing to >620 000 deaths among Indians annually.^1^ As per recent estimates from Global Burden of Disease (GBD), 2017, 10.6% of total deaths, 6.4% of total disability-adjusted life years, and 30% of cardiovascular deaths in India are attributable to ambient PM_2.5_ exposures.^2^ The annual population-weighted mean exposure to ambient particulate matter PM_2.5_ in India was 89.9 µg/m^3^ (95% confidence interval [CI]: 67.0-112.0) in 2017,^1^ far exceeding the World Health Organization (WHO) target of 10 µg/m^3^.^3^ Due to the limited spatial coverage of ground-level monitoring stations, there has been an increasing interest in the use of alternative methods to predict air pollution exposures in India.

The majority of evidence of health effects of air pollution has come from developed countries which does not account for the complex rapid urbanization happening in developing countries. Moreover, the composition of air pollution is very different in low- and middle-income countries (LMICs), and the health impact is influenced by several other meteorological, demographic, and built environment factors. The health impacts of air pollution are myriad and include respiratory and cardiometabolic diseases to reproductive disorders and infant morbidity.^4^ After respiratory disorders, cardiometabolic disorders are the most important diseases that are attributable to exposure to ambient air pollution,^5^ specifically PM_2.5_.^6^ Prospective cohort studies are very useful for studying multiple outcomes and are among the strongest designs for evaluating causal effects outside of randomized controlled trials. While several cohort studies exist in India, they have largely focused on pregnancy and birth outcomes. There are very few well-phenotyped adult cohorts focused on cardiometabolic health that are now beginning to study the impact of environmental risk factors on chronic diseases.

Considering that India is home to one-fifth of the world’s population, studies specific to the Indian scenario are urgently needed to shift policy discourse around ambient air pollution. Findings from a large multicountry study reported that stroke and ischemic heart disease (IHD) were 2 largest contributors for premature deaths and accounted for 74% of the total premature deaths in South and South-East Asia,^7^ with India contributing the most premature deaths of any country in the region. To date, only 1 study has evaluated the impact of air pollution on cardiometabolic disease outcomes in India: a time-series study in Varanasi that found that the achievement of the WHO air quality standard would prevent 1900 premature deaths every year.^8^ With improved air quality, modeled data estimates indicate that 24.0% of IHD and 18.5% of stroke deaths in India could be averted.^9^

The overall goal of the present GEOHealth (Global Environmental and Occupational Health) India Hub program is to leverage an ongoing cohort study in India, the Centre for cArdiometabolic Risk Reduction in South-Asia (CARRS) surveillance study,^10^ to evaluate the prospective effects of ambient air pollution on cardiometabolic health outcomes and associated traits. The study also leverages publicly available information on air quality, meteorological variables, and other environmental factors (like land use and emission inventories) for the analysis of complex spatiotemporal data and multipollutant exposures, and can serve as a proof of concept for other ongoing cohort studies in India and around the world. The analysis is facilitated by previously developed and validated prediction models for PM_2.5_ in the United States that combine land-use regression (LUR) with satellite-derived aerosol optical depth (AOD) data to estimate particle exposure.^11^

In addition, we are building on existing laboratory capacity in India to explore factors that may mediate the association between air pollution and cardiometabolic disease such as DNA methylation and vitamin D levels. Methylomics provides a unique opportunity to reconstruct past exposures, particularly those such as air pollution and other exposures that augment oxidative stress, to which the methylome is exquisitely sensitive.^12^ Air pollution in particular is known to alter DNA methylation that in turn is known to be associated with cardiometabolic health outcomes.^13-15^ Similarly, there is strong evidence on effect of Vitamin D levels and deficiency with various cardiometabolic outcomes.^16-21^ Particulate air pollution reduces effective UV-B exposure, which is critical for producing the biologically active form of vitamin D, and contributes to variation in the UV index, along with other factors such as latitude, season, skin pigmentation, and the use of sun-protective wear.^22,23^

Similar to this context, the GBD estimates emerge from a low-resolution chemical transport model that estimates particulate matter levels with considerable error and exposure-response functions based largely on research from low- and mid-level exposure settings.^24^ Most of the air pollution studies in LMICs (largely represented by China) are time-series/ecological study design with a short observation period which often has no spatiotemporal resolution of pollution parameters and focuses only on short-term health outcomes.^25^ Hence, this research will provide crucial, country-specific evidence of the health effects of air pollution to provide evidence for appropriate policy reforms. This study will also demonstrate how existing cohorts with longitudinal information on cardiometabolic health can be used to understand emerging risk factors and provide timely scientific data to inform cardiometabolic disease prevention and air pollution mitigation policies.

## CARRS Cohort

The CARRS surveillance study is a hybrid cohort-modeled cross-sectional study involving a baseline survey followed by repeat surveys carried out in subsequent years with a response rate of approximately 85%. The CARRS participants were recruited at baseline in 2010-2012 (Cohort 1) from 3 urban sites, Delhi and Chennai in India and Karachi in Pakistan. Thereafter, more participants were recruited from these cities in 2014-2016 (Cohort 2) to achieve larger sample size to understand the incidence of cardiometabolic risk factors, diseases, comorbidities, and mortality^10^ in this south-east Asian region.

Households were selected in each of the 3 cities using a multistage cluster random sampling technique from each ward and census enumeration blocks. Two participants, 1 male and 1 female, aged 20 years or older and permanently residing in the household, were selected from each household using “Kish method” used in the WHO’s (World Health Organisation stepwise Approach to Surveillance) (STEPS) surveys. Pregnant women and bed-ridden individuals were excluded from the study, and information on basic demographic details of these excluded individuals was recorded along with non-participating eligible participants. To provide consistency and reproducibility of the results across multiple sites and across different follow-ups, comprehensive and uniform data collection instruments were used to capture measurements. The details of all the data collection and study procedures have been described previously.^10^

The CARRS participants (Table 1) were phenotyped for a range of cardiovascular diesease (CVD) risk factors at baseline. Thereafter, every year these participants are being followed for CVD events and additionally for lifestyle factors, physical examinations, and biological samples as well in every alternate year (Table 2). This intense phenotype and built environment data are integrated into a Geographical Information System (GIS)–linked database. The data on geocoded residence of the participants and how long they have lived at their present location provide an excellent opportunity to estimate air pollution exposure levels. As far as possible, we are also trying to gather information on migration and also geocode current residence of the participants, if they have migrated within the cities. Written informed consent was obtained from CARRS participants to utilize their de-identified phenotype data and stored de-identified biological samples for future cardiovascular research.

**Table 1.** Baseline Characteristics of CARRS cohort.

Variables	Categories	CARRS-I			CARRS-II		
		Chennai	Delhi	Total	Chennai	Delhi	Total
N		6906	5364	12 270	4866	4725	9591
Age, mean (SD)		41.4 (12.7)	44.4 (13.5)	42.9 (13.1)	43.6 (13.1)	45.0 (13.8)	44.3 (13.4)
Gender							
Male		3188	2680	5868	2247	2243	4490
Female		3718	2684	6402	2619	2482	5101
Occupation							
Not working		3502	2754	6256	2296	2717	5013
Semi-/unskilled		1757	971	2728	1333	661	1994
Trained/skilled		1539	1342	2881	1160	1247	2407
White collar		108	297	405	77	100	177

Abbreviations: CARRS, Centre for cArdiometabolic Risk Reduction in South-Asia; SD, standard deviation.

**Table 2.** Summary of data collected over time in CARRS cohort study.

Cohort	N	Available data^a^
CARRS-I		
Baseline (2010-2012)	12 271	• Sociodemographic indicators^b^ • Socioeconomic status^b^ • Health expenditure • Lifestyle risk factors such as tobacco and alcohol use, diet, and physical activity • Body fat distribution (anthropometry and bioelectric impedance) • Self-reported and incident hypertension, diabetes, stroke, MI, other cardiovascular diseases (CVD), CKD^c^ • Serological and biochemical parameters such as lipids and glycemic traits, LFT, KFT, inflammatory markers, cotinine levels • ECG data (only for Delhi site)^d^ • Biorepository for future studies (DNA, serum, plasma, urine, buffy coats, etc)
1st FUP (2011-2013)	9194	
2nd FUP (2013-2014)	9619	
3rd FUP (2014)	8115	
4th FUP (2016-2017)	7372	
5th FUP (2017-2018)	6969	
CARRS-II		
Baseline (2014-2016)	9594	
1st FUP (2018-2020)	Ongoing	

Abbreviations: CARRS, Centre for cArdiometabolic Risk Reduction in South-Asia; CVD, cardiovascular disease; CKD, chronic kidney disease; ECG, electrocardiogram; FUP, follow-up; KFT, kidney function test; LFT, liver function test; MI, myocardial infarction

a The detailed phenotyping including CVD risk factors, physical measures, and biological samples was taken at baseline and 2nd and 4th follow-ups. Events and cause of death data were captured only in 1st, 3rd, and 5th follow-ups.

b Available for baseline and 4th follow-up.

c Events and cause of death available at all time points.

d Available at 2nd and 4th follow-ups.

The GEOHealth program is utilizing information from the Delhi and Chennai sites having a very different cardiometabolic profile along with different geospatial determinants and air pollution levels and composition. We are restricting our GEOHealth proposed objectives to only Indian cities for logistic and feasibility purposes around estimating ambient air pollution exposure levels.

## Research Aims

### Aim 1: Estimate air pollution exposure in Chennai and Delhi at fine spatiotemporal resolution

We will develop and validate exposure models to estimate daily exposure to fine particulate matter (PM_2.5_) at a 1 km × 1 km spatial resolution from 2010 to 2016.^26^ The predicted concentrations will be used to assign ambient air pollution exposure values to >15 000 CARRS households in Chennai and New Delhi. The prediction models are based on machine learning methodologies and ensemble averaging while using ground monitoring data, satellite measurements, meteorological data, land-use variables, and emission inventories.^11^ The major advantage of this modeling exercise is that it enables us to obtain neighborhood-level ambient concentrations irrespective of the presence or absence of the monitoring network. In addition, the fine spatiotemporal resolution of the exposure enables us to estimate effects on health outcomes at an individual level over time. The future aim is to extend this model across all of India as well as over longer periods of time and also for other pollutants, including NO_2_ and ozone.

### Aim 2: Estimate the association between exposure to air pollution, temperature, cardiometabolic diseases, and risk factors, and identify potential susceptible subpopulations

We aim to estimate that the association of ambient air pollution exposure from Aim 1 is within the CARRS cohort. In addition to estimating main effects, we will evaluate effect modification by population subgroups, based on their socioeconomic status, built environment, occupational status, and nutritional status to identify those most susceptible groups. The minimum detectable extreme quartile relative risks for 80% power with a 5% Type I error rate were calculated for the CARRS-1 cohort in Delhi and Chennai (n = 12 271) using the observed 2- and 3-year follow-up rates. To assess power of this study to detect correlations in prospective changes in markers of cardiometabolic (CM) risk such as HBA1c, lipid profiles, serum creatinine, and blood pressure, we find that we will have 80% power to detect correlations as low as 2% to 3% longitudinally as well as cross-sectionally with baseline air pollution (AP) constituents, given the baseline sample size and observed follow-up rates at 2 and 3 years.^27^

### Aim 3: Characterize DNA methylation patterns–associated cardiovascular events and explore whether DNA methylation mediates the association between air pollution exposure and cardiovascular outcomes

Given the limited sample size and budget, the methylomics aim will focus on cardiometabolic outcomes through a nested case-control design (approximately n = 192 cardiovascular events and controls [myocardial infarction/strokes or CVD deaths) to explore whether methylomic patterns mediate the effect of PM_2.5_ exposures on CVD events using mediation analyses; 96 cases and controls will provide 98% power to detect 5% methylation difference between the groups assuming a conservative standard deviation (SD) of 5% in each group at *P* = 1.1 × 10^−6^.

### Aim 4: Explore the association between ambient exposure to air pollution and blood vitamin D levels

We will explore associations between air pollution exposure and blood vitamin D levels (measured as 25-OH-D levels), and we will examine whether vitamin D levels are a mediator of the association between air pollution and cardiometabolic outcomes using causal mediation analysis. We will randomly sample 600 CARRS participants from Delhi and Chennai who will provide 80% power or more to detect correlations as low as 2% to 3% longitudinally as well as cross-sectionally at baseline.

The detailed analysis approach for all the 4 research aims is described in Table 3.

**Table 3.** Proposed analysis approach for the research aims.

Research aims	Analysis approach
Aim 1: Estimation of air pollution exposure	A 3-stage hybrid model utilizing machine learning algorithms, ensemble averaging, and tensor product smoothing
Aim 2: Estimation of the association between air pollution exposure and cardiometabolic disease risk factors and diseases	• Longitudinal mixed effects modeling • Time to event modeling for incidence data
Aim 3: Characterization of DNA methylation patterns associated cardiovascular health effects and cardiometabolic (CM) outcomes	• Linear regression analyses to identify the DNA methylation profile associated with cardiovascular disease (CVD) events • Mediation analyses to explore whether these associations are mediated by PM_2.5_ levels
Aim 4: Estimation of the association between air pollution exposure and blood vitamin D levels	Mediation analyses to examine the mediating effect of vitamin D in association between PM_2.5_ and cardiovascular disease (CVD)

## Capacity Building

One of the major goals of this study is to build training and research capacity to address environmental and occupational health research questions beyond the specific aims of this grant. We have laid out multiple different ways to achieve this: faculty from the Harvard T.H. Chan School of Public Health (HSPH) will collaborate and train investigators from Centre for Chronic Disease Control (CCDC) and Public Health Foundation of India (PHFI):

- Mentored training program wherein the researchers from PHFI and CCDC will work and learn along with an identified mentor at HSPH.
- Summer exchange visits at HSPH, to further strengthen capacity of the researchers from PHFI and CCDC.
- Master’s training program (fully sponsored MPH or MSc in environmental health at HSPH).
- PHFI and CCDC are running 5-day short courses to complement research activities. Courses on introduction to environmental health, research ethics in environmental health, air pollution epidemiology, food and the environment, principles of toxicology, environmental exposure assessment, occupational health and medicine, causal modeling, and air pollution, climate, and health: modeling and methods have been conducted. The design of the courses is such that in the first couple of years, HSPH faculty will lead the course and PHFI and CCDC faculty can take lead thereafter.
- HSPH along with faculty from PHFI and CCDC are working together in developing curriculum for MPH in environmental health track in India.

## Innovation

The innovation of our study lies in the methodology of exposure assessment and the estimation of health effects in a cohort study with longitudinally measured health outcomes in 2 major Indian cities. To date, ambient air pollution exposure assessment in India has been reliant on source apportionment, emission inventories, satellite remote sensing, and LUR techniques. Due to inherent limitations of each methodology, the exposure estimates are often coarse in spatial resolution and/or fail to capture temporal variability. The methodology used in this study incorporates the strengths of multiple machine learning techniques along with the most relevant sources of data, thus providing high resolution on both spatial and temporal scales. To the best of our knowledge, the GEOHealth study is the first to assess the effects of ambient air pollution on multiple incident cardiometabolic disease and associated risk factors in India with one of the highest ambient levels of PM_2.5_ in the world. Our results will also help in understanding the complex interplay of the role of the air pollution, the built environment, occupational exposure, and sociodemographic factors on cardiometabolic risk factors in India which is facing major development and epidemiological transitions.

Air pollution modeling work that will be undertaken in this project will improve upon the GBD estimates. This exposure assessment approach is much more rigorous and comprehensive, with a fine spatiotemporal resolution of 1 km by 1 km compared with GBD resolution of 11 km by 11 km.^24^ This can serve as a resource even for other health outcomes in that space-time boundary. In addition, we have individual-level information on health and other variables that help in providing more reliable exposure-response curves.

## Impact

Through this GEOHealth Hub, we are enhancing research activities and providing scientific infrastructure, training, and capacity building to characterize the relationship between air pollution and cardiometabolic risk factors and diseases in India. This is the largest and most extensive effort to address this issue in India, an LMIC with very high air pollution levels and prevalence of CM risk factors. The study is expected to produce results that will (1) advance the science regarding exposure assessment and effects of air pollution on CM risk factors; (2) inform urban planning and transportation planning policies designed to improve health in India, while taking into account air pollution exposures; (3) contribute important information to the gap in knowledge on the environmental contributions to CM risk factors and how effects are mediated by vitamin D levels and epigenetic mechanisms; and (4) inform development and implementation of targeted regulations, policies, and interventions to promote healthier living in India. In addition, this research can serve as a template for developing national-level pollution models, which can be further used to study the effects of pollution on diverse health outcomes.
